# Improving Sleep with Far-Infrared-Emitting Pajamas: A Pilot Randomized Controlled Trial

**DOI:** 10.3390/ijerph20053870

**Published:** 2023-02-22

**Authors:** Shu-Cheng Chen, Tin-Wai Cheung, Branda Yee-Man Yu, Mei-Yan Chan, Wing-Fai Yeung, Li Li

**Affiliations:** 1 School of Nursing, The Hong Kong Polytechnic University, Hong Kong SAR 999077, China; 2 School of Fashion and Textiles, The Hong Kong Polytechnic University, Hong Kong SAR 999077, China; 3 Department of Psychology, The University of Hong Kong, Hong Kong SAR 999077, China

**Keywords:** far infrared-emitting pajamas, RCT, sleep disorders, adults

## Abstract

Far infrared (FIR)-based clothing may alleviate sleep disturbance. This study aimed to explore the effects of FIR-emitting pajamas on sleep quality. This was a pilot randomized, sham-controlled trial. Forty subjects with poor sleep quality were randomized to FIR-emitting-pajamas and sham-pajamas groups in a 1:1 ratio. The primary outcome measure was the Pittsburgh Sleep Quality Index (PSQI). Other measures included the Insomnia Severity Index, and 7 day sleep diary, the Multidimensional Fatigue Inventory (MFI), the Hospital Anxiety and Depression Scale, the Epworth Sleepiness Scale, and the Satisfaction with Life Scale. Outcomes were measured at baseline and weeks 2, 4, and 6. Both groups showed within-group improvements in the PSQI score, but there was no significant difference between the two groups. However, FIR-emitting pajamas appeared to perform better than sham pajamas in reducing the MFI-physical score, with large effect sizes at three time points (dppc2 = 0.958, 0.841, 0.896); however, the differences were statistically insignificant. The intervention compliance was satisfactory. The effects of FIR-emitting pajamas on sleep quality were not superior to those in the control group. However, these pajamas may improve physical fatigue in adults with poor sleep quality, which warrants further exploration.

## 1. Introduction

Self-reported sleep difficulties are a highly prevalent health complaint, with a range of 10–48% of the general population worldwide experiencing sleep problems accompanied by daytime consequences [1,2,3]. Individuals with sleep problems may complain of insomnia symptoms, such as difficulties initiating sleep, difficulties maintaining sleep, and/or early morning awakening [4]. If the insomnia symptoms are sufficiently frequent and chronic and lead to substantial distress and impairments to daytime functioning, the sleep problems can be classified as insomnia disorder [5]. In China, the prevalence of insomnia determined using a sleep questionnaire cut-off score was 15% [6]. In Hong Kong, the prevalence of sleep disturbance ranged from 19.9% to 39.4% [7,8,9,10], and 40.1% of Hong Kong people reported at least one insomnia symptom three or more nights per week, with 10.8% fulfilling the insomnia disorder criteria [4]. Sleep deprivation leads to fatigue, irritability, impaired daytime functioning, and increased work absenteeism [11]. Poor sleep is also associated with increased risk of psychological disorders, suicide, cardiovascular disease, and headache [12,13]. Early diagnosis and treatment of sleep problems are crucial because, if left untreated, they may predispose individuals to developing anxiety and depressive disorders [14].

Sleep can be regulated by optimizing the environment in which one sleeps. This is achieved by controlling environmental factors, such as temperature, lighting, and noise [15]. In addition to regulating room temperature, clothes with thermoregulation functions may be an option. Clothing made from far infrared (FIR)-based textiles may be a possible material that could achieve such a purpose. Considering the bio-functions of FIR textiles, these materials have been shown to be capable of promoting blood circulation to body tissues and muscles because of the vasodilation caused by the energy absorption and transmission in human skin [16]. The improved blood circulation was verified to promote bio-healthcare abilities, such as keeping warm, increasing metabolic rate and blood oxygen level, and eliminating metabolic toxins [17,18]. In light of significant results regarding the enhancement of the radiation absorption efficiency and higher levels of thermal comfort, the proposed FIR pajama was expected to provide a new therapeutic alternative for poor sleepers. The developed textile apparel could alleviate sleep problems and the associated symptoms, enhancing daytime functioning, concentration, and subjective well-being in relation to sleep problems.

The effects of textiles embedded with FIR bio-ceramic technology on sleep have been examined in a pilot randomized controlled trial (RCT) by McCall and colleagues [19]. The results showed that sleeping on FIR-emitting bedsheets was associated with fewer reported insomnia symptoms than sleeping on a placebo sheet among 29 healthy adults [19]. However, the primary data were only established for healthy subjects, and only the Insomnia Severity Index was adopted for sleep evaluation. Further research with more standard sleep measures, such as a prospective sleep diary, is needed.

**Figure 1:**
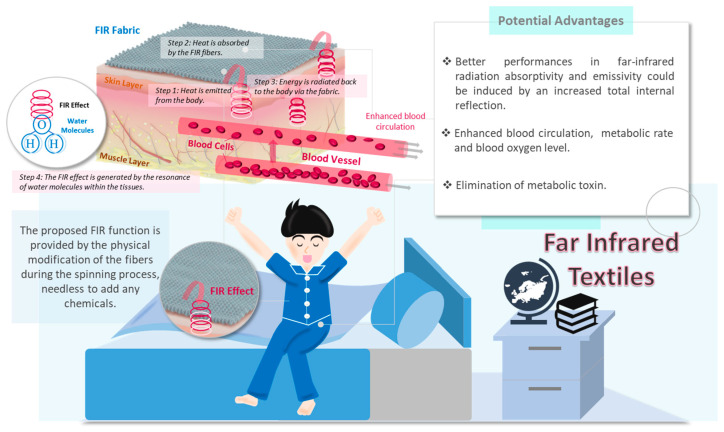
Schematic diagram of the far infrared textiles and the potential advantages.

Clothing made from FIR-based textiles could be a sustainable way to alleviate insomnia with many advantages. First, the proposed FIR textiles were composed of handmade profiled fibers without the addition of chemicals, such as ceramic additives or other related coatings. Enhanced FIR radiation absorptivity and emissivity were induced through the structural modification of the handmade fibers. Thus, the textiles were environmentally friendly and safe to wear. The pajamas made from FIR-based textiles thus had more advantages than bedsheets in relation to altering body temperature. Pajamas are worn on the body and are directly in contact with the skin. Hence, the far-infrared radiation absorptivity and emissivity, which were induced by the total internal reflection and the waves between the textiles and the skin, were more direct. This comfortable thermal environment was associated with better sleep quality. Furthermore, FIR-emitting pajamas, as an intervention with lower costs and requiring zero therapist input, can ensure longer-term sustainability of benefits compared to the traditional therapist-delivered treatment modality or self-help interventions demanding continuous effort (Figure 1). This is because FIR textiles can absorb heat radiation from the body and then emit it back, resulting in enhanced body temperature. The thermal effect in deep tissues can lead to vasodilation and promote blood microcirculation [16]. Therefore, improved thermoregulation (maintenance of internal and external body temperature) with greater tolerability and thermophysiological comfort can be achieved. In addition, these textiles facilitate sleep by creating a microclimate of warmth around the body that promotes relaxation and reduces muscle tension to help the body prepare for sleep. Given these advantages, we developed pajamas made from FIR-based textiles with a high level of thermal comfort to enhance sleep quality in subjects with sleep problems. To fill the research gap relating to this topic, we conducted a pilot RCT to preliminarily explore the effects of FIR energy on sleep by comparing specially designed pajamas to a sham-pajamas group.

The specific aims of this study were as follows: (1) to evaluate the short-term effects of FIR-emitting pajamas in relation to improving insomnia symptoms compared to the sham-pajamas control group, for which we measured sleep quality using the Pittsburgh Sleep Quality Index (primary outcome) and other sleep measures; and (2) to evaluate the acceptability of using FIR-emitting pajamas to enhance sleep quality.

## 2. Materials and Methods

### 2.1. Study Design

The study was a pilot randomized, parallel-group, double-blind, and sham-controlled trial on the effects of FIR energy on sleep disturbance using specially designed pajamas. Informed consent was obtained before all study procedures. Subjects were randomly assigned to the FIR-emitting pajamas (FIR pajamas) group or the sham-pajamas control group in a 1:1 ratio. Ethics approval was obtained from the local ethic review board (HSEARS20201109005). The trial was registered on ClinicalTrials.gov (ClinicalTrials.gov identifier: #NCT04890002), and all the study procedures followed the protocol in the register. This RCT was conducted and reported following CONSORT recommendations.

### 2.2. Subjects and Randomization

Subjects with insomnia symptoms were recruited using recruitment posters through universities and social network agencies from July 2021 to February 2022. Subjects were considered eligible if they: (1) were Chinese Hong Kong residents who could read Chinese; (2) were aged 18–65 years; (3) reported subjective sleep complaints relating to difficulties falling asleep, difficulties staying asleep, or early morning awakening with consequences for daily life for at least three months; (4) demonstrated Pittsburgh Sleep Quality Index (a) total scores of at least 5, indicating sleep disturbance with 89.6% sensitivity and 86.5% specificity (b); and (5) were willing to give informed consent and comply with the trial protocol.

Subjects were excluded if they: (1) were receiving psychotherapy or participating in other clinical trials for insomnia; (2) were pregnant; (3) were shift workers; (4) were at significant risk of suicide as rated by the Hamilton Depression Rating Scale item on suicide (score ≥ 3) [20]; (5) had no self-reported comorbid sleep disorders primarily requiring other treatment, such as sleep apnea or narcolepsy; and (6) were taking herbal remedies, over-the-counter medication, or psychotropic drugs that target insomnia within the two weeks before baseline.

A computer-generated randomization list with a random block size of four to six was prepared by an independent researcher. After the completion of baseline assessment, the independent researcher gave the group allocation to a staff member responsible for keeping the pajamas and delivering them to the subjects. All the assessments in this study were self-completed. The researchers who performed the assessment and analysis were blinded to the group allocation. All the subjects were blinded to their group allocation.

### 2.3. Interventions

**Figure 2:**
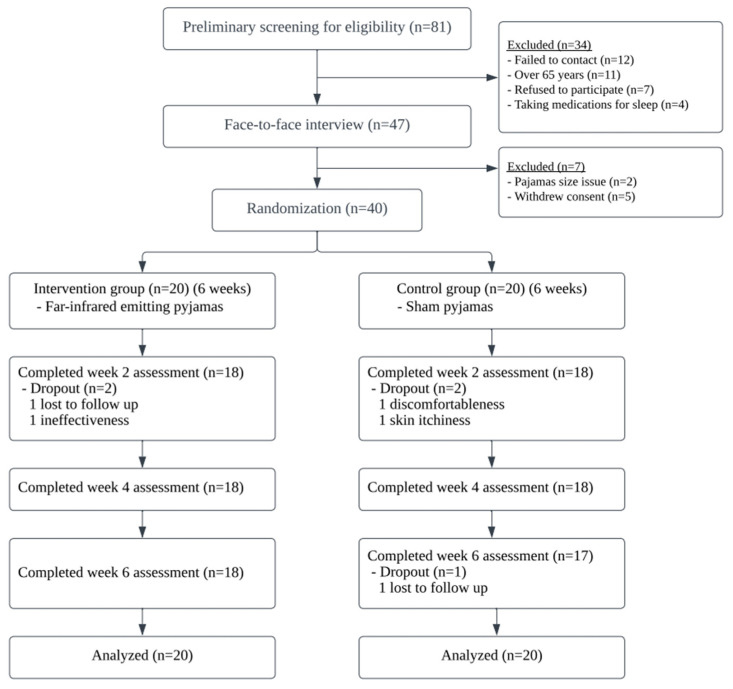
The CONSORT flowchart.

Eligible subjects’ body sizes were measured during the face-to-face screening. The pajamas were prepared in small, medium, large, and extra-large sizes (Figure 2). The subjects were randomized into either the FIR-pajamas group or the sham-pajamas control group in a 1:1 ratio.

#### 2.3.1. FIR-Emitting-Pajamas Group

Subjects in this group were provided with two sets of FIR-emitting pajamas. The FIR pajamas were fabricated using two textile materials: pure cotton fibers and the proposed handmade FIR fibers with the FIR-emitting function. The proposed FIR function was provided via the physical modification of the fibers during the spinning process without having to add any chemicals. In addition, based on the theoretical knowledge and simulations, it was believed that better performances in far-infrared radiation absorptivity and emissivity would be induced due to the increased total internal reflection, as well as the transmission of fewer infrared waves within the cross-sections. To ensure the quality of the proposed pajamas with FIR features, their performance in terms of FIR emissivity and temperature differences was evaluated and ensured in a certified testing lab based on the national standard method (GB/T 30127–2013) and requirements (the temperature difference had to not be less than 1.4 °C and the emissivity had to not be less than 0.88).

#### 2.3.2. Sham-Pajamas Group

To control for the placebo effect in the FIR-pajamas group, subjects in the sham-pajamas group received two sets of pajamas with identical physical appearances that were produced using the same fabrication process as the pajamas received by the FIR-pajamas group. The sham pajamas were made of pure cotton fibers and handmade fibers without the FIR-emitting function. The subjects were asked to wear the sham pajamas daily at night for six consecutive weeks.

### 2.4. Monitoring of Compliance at Home

Subjects were required to fill in a logbook to record the wearing of the assigned pajamas every night during the six-week study period. Previous studies used self-report logs to record self-help interventions, such as group cognitive behavioral therapy, self-acupressure, and exercise [21,22,23,24,25,26]. The research assistant sent weekly message reminders during the six-week study period to remind subjects to wear the assigned pajamas before bedtime, answer questions, and provide feedback regarding the FIR-emitting features. The introduction of any new sleep-focused treatment during the study period was not allowed.

### 2.5. Outcome Assessments

#### Patient-Centered Outcome Assessment

Patient-centered outcome assessments were conducted at four time points: baseline, week 2, week 4, and week 6. The primary outcome was the Pittsburgh Sleep Quality Index (PSQI) [27], which was assessed at baseline and weeks 2, 4, and 6. The PSQI is a self-reported evaluation of overall sleep quality. Subjects were asked to report the sleep parameters of their sleep problem, use of sleep medicine, disturbances, and daytime dysfunction with ratings of 0–3. The total score for the PSQI ranges from 0 to 21, and a scores ≥ 5 indicates poor sleep quality. The time frame adopted for the PSQI in this study was changed from “past one month” to “past two weeks”. The PSQI is a reliable and validated tool widely used in sleep studies. Other secondary assessments included the Insomnia Severity Index (ISI) for the severity of insomnia symptoms and related daytime impairment (the total score ranges from 0 to 28, and a score ≥ 15 indicates moderate or severe insomnia) [28], the Hospital Anxiety and Depression Scale (HADS) for the severity of depression and anxiety (the total score ranges from 0 to 21, and a score of 11–21 indicates depression or anxiety) [29], the 20 item Multidimensional Fatigue Inventory (MFI-20) for fatigue level, the Epworth Sleepiness Scale (ESS) for the level of daytime sleepiness in eight common daily activities (the total score ranges from 0 to 24, and a score of 11–24 indicates excessive daytime symptoms) [30], and the five-item Satisfaction with Life Scale (SWLS) to measure individuals’ experiences of life satisfaction (the total score ranges from 5 to 35, and a score of 5–20 indicates dissatisfaction with life) [31]. A 7 day sleep diary was used to record subjects’ sleep parameters daily for seven days before baseline and in week 6 [32,33]. Subjects were told to record their daily bedtime and waking time, from which the total time in bed (TIB) was calculated. Subjects were asked to estimate their sleep onset latency (SOL), wakefulness after sleep onset (WASO), and total sleep time (TST), from which sleep efficiency (SE) was then calculated as TST/TIB × 100%.

### 2.6. Adherence Assessment

To assess their adherence to the use of the assigned pajamas, participants were given a logbook to record their wearing of the assigned pajamas during the six-week study period. The total number of days when the pajamas were worn was counted. Subjects were considered non-adherent if they missed more than two nights in a week. The participants were provided with free pajamas but did not obtain monetary compensation or other awards for adhering to the intervention.

### 2.7. Sample Size Estimation

For the pilot study, a sample of at least 15 was suggested to ensure sufficient methodological experience and a reasonable estimation of the preliminary effect size required to conduct a fully powered study [34,35]. Considering a dropout rate of 25% at the six-week post-intervention follow-up, 20 subjects per group were needed.

### 2.8. Data Management and Analysis

All data were double entered for consistency prior to analysis. An intention-to-treat analysis was employed. All statistical analyses were performed in SPSS for Windows. Changes in PSQI scores (primary outcome), ISI scores, subjective sleep parameters, and other questionnaire scores compared to baseline were examined using the GEE with repeated measures and dropout considered by including all available data points (intent-to-treat analysis). Groups and time points were fixed factors, and subjects were the random factor. Significant time-by-group interactions supported our hypothesis on the effects of FIR energy on sleep. Time and group interaction effect sizes were calculated based on the mean pre–post change in the treatment group minus the mean pre–post change in the control group divided by the pooled pretest standard deviation [36]. A completer analysis of the primary outcome measure was performed for those who adhered to the intervention by wearing the pajamas for at least 36 days during the study period.

## 3. Results

### 3.1. Characteristics of Recruited Subjects

A total of 40 subjects were randomized. The CONSORT diagram is shown in Figure 2. Table 1 shows that the recruited subjects were mainly female (85.0%), with a mean age of 51.23 years (SD = 11.212) and a BMI of 22.361 kg/m^2^ (SD = 4.481). The mean score for the PSQI was 9.33 (SD = 3.058). There were no significant differences in demographic characteristics and baseline assessments between the two groups.

### 3.2. Intervention Compliance

Two and three subjects dropped out in the intervention and control groups, respectively (12.5%). In this study, an intervention completer was defined as a participant that wore the pajamas for 36 days or more. During the 42 day study period, 35 subjects (87.5%, 19 in the intervention group and 16 in the control group) wore the pajamas for 36 days or more and 24 subjects (60%, 14 in the intervention group and 10 in the control group) wore the pajamas every night. There were no significant differences between the two groups in the numbers of days pajamas were worn, indicating adherence (*p* = 0.11).

### 3.3. Primary Outcome

The FIR-pajamas and sham-pajamas groups showed non-significant improvements in PSQI scores at all three time points (FIR pajamas: Cohen’s d for week 2, week 4, and week 6 = 0.15, 0.364, and 0.356; sham pajamas: Cohen’s d for week 2, week 4, and week 6 = 0.655, 0.78, and 0.754; all *p* > 0.05) (Table 2). Compared to the FIR-pajamas group, the sham-pajamas group showed a statistical trend toward greater reductions in PSQI scores with a small effect size at all three time points (week 2: dppc2 = 0.393, *p* = 0.112; week 4: dppc2 = 0.328, *p* = 0.273; week 6: dppc2 = 0.231, *p* = 0.255). Completer analyses revealed similar findings for PSQI score changes (Supplementary Materials).

### 3.4. Secondary Outcomes

#### 3.4.1. ISI

The FIR-pajamas and sham-pajamas groups showed non-significant improvements at all three time points (FIR pajamas: Cohen’s d for week 2, week 4, and week 6 = 0.271, 0.749, and 0.881; sham pajamas: Cohen’s d for week 2, week 4, and week 6 = 0.598, 1.051, and 0.709; all *p* > 0.05, Table 2). The effect sizes for the differences between the two groups were small across the assessment time points (week 2: dppc2 = 0.357, *p* = 0.167; week 4: dppc2 = 0.353, *p* = 0.254; week 6: dppc2 = 0.033, *p* = 0.917).

#### 3.4.2. The Seven-Day Sleep Diary

Both groups showed small or moderate within-group effect sizes for improvements at week 6 in the sleep diary-measured SOL (FIR pajamas: Cohen’s d for week 6 = 0.181; sham pajamas: Cohen’s d for week 6 = 0.344; *p* = 0.565), WASO (FIR pajamas: Cohen’s d for week 6 = 0.666; sham pajamas: Cohen’s d for week 6 = 0.589; *p* = 0.176), TST (FIR pajamas: Cohen’s d for week 6 = 0.398; sham pajamas: Cohen’s d for week 6 = 0.505; *p* = 0.852), and SE (FIR pajamas: Cohen’s d for week 6 = 0.308; sham pajamas: Cohen’s d for week 6 = 0.395; *p* = 0.648). No significant between-group differences were observed in the sleep parameters recorded in the sleep diary at any of the study visits (Table 2), but the FIR-pajamas group showed a trend toward improvement over the sham pajamas group in WASO with a small effect size at week 6 (dppc2 = 0.327, *p* = 0.176).

#### 3.4.3. HADS, MFI, Satisfaction with Life Scale, ESS

Table 3 shows that there were no significant between-group differences in HADS anxiety and depression scores; Satisfaction with Life Scale scores; or MFI-general, MFI-mental, MFI-activities, or MFI-motivation scores. The FIR-pajamas intervention performed better than the sham pajamas in reducing the MFI-physical score across all the time points with large effect sizes (week 2: dppc2 = 0.958, *p* = 0.031; week 4: dppc2 = 0.841, *p* = 0.075; week 6: dppc2 = 0.896, *p* = 0.123). The sham-pajamas group showed a greater improvement in the ESS score compared to the FIR-pajamas group with small to moderate effect sizes at weeks 2 and 4 (week 2: dppc2 = −0.495, *p* = 0.046; week 4: dppc2 = −0.556, *p* = 0.041).

#### 3.4.4. Blinding

The subjects were asked to guess their group allocations (FIR pajamas, sham pajamas, or no idea). Although the proportions of subjects with no idea which pajamas they had received were similar (25% vs. 26.3%), a significantly higher proportion of subjects in the intervention group guessed that they had received FIR pajamas (60.0% vs. 15.8%, chi-squared test, *p* = 0.007).

#### 3.4.5. Adverse Events

In the FIR-pajamas group, one subject perceived that the FIR pajamas were ineffective for him and then dropped out during the second week. In the sham-pajamas group, one subject felt uncomfortable when putting on the pajamas and withdrew during the second week; another subject suffered from skin itchiness after wearing the pajamas and dropped out during the second week. Both groups had one subject lost to follow-up without reasons being given.

#### 3.4.6. Post Hoc Power Analysis

The post hoc power analysis revealed that the powers of the present sample size in determining the differences between MFI-physical scores at week 2 (effect size = 0.958), week 4 (effect size = 0.841), and week 6 (effect size = 0.896) were 83.9%, 73.6%, and 78.8% respectively. The effect size estimated in this study suggested that a sample size of 24 for each group would be needed to detect between-group differences in MFI-physical scores at week 4 with a power of 80% and type I errors at 5%.

## 4. Discussion

To the best of our knowledge, the present study was the first RCT to examine the effects of FIR-emitting pajamas on sleep in adults with sleep disturbance. The present study showed that the subject recruitment and intervention delivery were feasible. The compliance to intervention was satisfactory, with 35 subjects (87.5%) wearing the pajamas for 36 nights or more. Both FIR-emitting and sham pajamas showed a trend of improving sleep quality, mood, or anxiety symptoms, and the differences between the two were small. Insignificant improvements in physical fatigue and daytime sleepiness were observed in the intervention group compared to the sham-pajamas group, and better MFI-physical scores were recorded for the intervention group specifically. The FIR pajamas were generally safe, as only one subject dropped out in the intervention group because of personal perceived ineffectiveness.

The results revealed a non-significant improvement in sleep quality in the sham-pajamas group, who wore pajamas made of ordinary cotton and handmade fibers, but not in the FIR-pajamas group. The improvements observed in the sham-pajamas group can be attributed to the normal progression of the disease, the placebo effects of the sham intervention, and regression to the mean [37]. Psychological effects might also have relationships with sleep rituals [38]. Donning of the pajamas during the research period may have been treated as a contemporary sleep ritual by the subjects, becoming a routine before going to bed. The subjects may have had expectations leading them to relax and wind down after putting on the pajamas, and it may also have been a signal to get rest for the brain [39]. Such expectations may also have strengthened the association with bedtime as a proxy of the sleep intervention [39].

The sham-pajamas group showed nearly a two-point improvement in the PSQI score and a four-point improvement in the ISI score. The improvements observed in the sham control group can be explained by the normal progression of insomnia and non-specific effects associated with the sham pajamas. First, insomnia symptoms can be self-remitting. A previous local longitudinal study found that as large a proportion as 55.8% of individuals suffering from insomnia experienced remittal at the 1-year follow-up [40] Second, the sham pajamas might have resulted in improvements in the participants due to the non-specific effects of the pajamas, such as those relating to wearing pajamas made of high-technology clothing material, putting on the pajamas as a sleep ritual, and the participants’ psychological states. One of the possible explanations is that the infrared pajamas kept the body warm, which may have helped the body temperature adjust to a comfortable level that facilitated the initiation or re-initiation of sleep [41]. Cotton cloth materials may be more comfortable for the Hong Kong environment, which is usually warm and highly humid.

Though statistically insignificant improvements in physical fatigue and sleepiness were observed, better performance in reducing MFI-physical scores, with large effect sizes at three time points (*d^ppc^*2 = 0.958, 0.841, 0.896), was found for those wearing the FIR pajamas compared to the sham-pajama group. The findings suggest that the infrared emissions may have potentially been effective in improving physical recovery and daytime sleepiness. Since the heat emitted from the human body can be further re-emitted in the form of FIR radiation, the proposed FIR fibers can induce greater total internal reflection and a higher optical path difference; hence, FIR radiation can both penetrate deeply into tissues and eliminate toxins via vibrational resonance through the stimulation of water molecules. Based on this phenomenon, the molecular kinetic energy can be increased, leading to increases in skin and muscle temperature, as well as the vasodilation of blood vessels. Previous studies found that FIR fibers can enhance blood circulation [16], metabolic rate and blood oxygen level, and elimination of metabolic toxins [17,18]. These functions may help to explain the improvement in fatigue and the reduced daytime sleepiness. Future trials could target those who experience physical fatigue and daytime sleepiness. The impact of the FIR-emitting pajamas on muscles and the lymphatic system might alleviate pain and function as a crucial non-pharmacological treatment [42]. The alleviation in pain might make it possible to disentangle the sleep–pain complex association. Further studies could explore the effects of FIR-emitting pajamas on pain in people with insomnia symptoms.

This study had several limitations in addition to its small sample size. First, this study did not use polysomnography to exclude those with other possible sleep disorders, such as sleep apnea and periodic limb movement disorder, which may also present with sleep complaints. Second, no objective sleep measures, such as actigraphy, were used as outcome measures. Nevertheless, essential subjective sleep assessments [33], including validated sleep questionnaires, such as the PSQI and ISI, and a seven-day prospectively recorded sleep diary, were adopted. Future studies should include objective sleep measures to corroborate the findings. Third, the sham pajamas did not successfully mask the group allocation. However, no significant differences were found for the between-group effect sizes observed, which indicated that the effects of the FIR pajamas were not seriously inflated by the relevant issues arising from blinding (e.g., the psychological effect on subjects). Fourth, 85% of the participants were female, which may restrict the external validity. Finally, we did not include a waitlist control group and, thus, could not quantify the placebo effect of the sham pajamas in sleep improvement.

## 5. Conclusions

The FIR-emitting pajamas were a generally safe intervention. Although the findings from the pilot RCT showed that the effects of the FIR-emitting pajamas on adults with poor sleep were not significant when compared to the sham control, their effects on physical fatigue could be explored further. Future studies could specifically examine their effects related to relieving fatigue.

## Figures and Tables

**Table 1:** Demographics and baseline characteristics of the two groups.

Variable ^a^	All Subjects (*n* = 40)	FIR-Pajamas Group (*n* = 20)	Sham-Pajamas Group (*n* = 20)	*p*-Value ^b^
Age (y)	51.23 (11.212)	53.10 (10.508)	49.35 (11.842)	0.296
Female gender (%)				0.376
Female	34 (85.0%)	16 (47.1%)	18 (52.9%)	
Educational level (y)				0.062
Primary education or below	1 (2.5%)	0 (0.0%)	1 (100.0%)	
S1–S3	4 (10.0%)	4 (100.0%)	0 (0.0%)	
S4–S7	13 (32.5%)	8 (61.5%)	5 (38.5%)	
Tertiary education or above	22 (55.0%)	8 (36.4%)	14 (63.6%)	
Marital status (%)				0.729
Single	8 (20.0%)	3 (37.5%)	5 (62.5%)	
Married	30 (75.0%)	16 (53.3%)	14 (46.7%)	
Divorced/widowed	2 (5.0%)	1 (50.0%)	1 (50.0%)	
Employment status (%)				
Employed	22 (55.0%)	11 (50.0%)	11 (50.0%)	
Unemployed/retired/housework	18 (45.0%)	9 (50.0%)	9 (50.0%)	1.000
BMI (kg/m^2^)	22.361 (4.481)	21.997 (2.755)	22.726 (5.774)	0.613
Hospitalization for physical disease (%)	12.0 (30.0%)	7 (58.3%)	5 (41.7%)	0.490
Previous intervention for sleep disturbance (%)	30 (75.0%)	15 (50.0%)	15 (50.0%)	1.000
Western medicine	3 (7.5%)	2 (66.7%)	1 (33.3%)	0.548
Chinese herbal medicine	13 (32.5%)	6 (46.2%)	7 (53.8%)	0.736
Acupuncture	10 (25.0%)	4 (40.0%)	6 (60.0%)	0.465
Massage	8 (20.0%)	5 (62.5%)	3 (37.5%)	0.429
Sports	18 (45.0%)	8 (44.4%)	10 (55.6%)	0.525
Psychotherapy	0 (0.0%)	0 (0.0%)	0 (0.0%)	1.000
Others	5 (12.5%)	3 (6.0.%)	2 (40.0%)	0.633
Pittsburgh Sleep Quality Index	9.33 (3.058)	9.50 (3.269)	9.15 (2.907)	0.722
Insomnia Severity Index	14.70 (4.490)	14.30 (4.231)	15.10 (4.811)	0.580
Seven-day sleep diary				
Sleep onset latency	38.575 (28.241)	36.614 (26.657)	40.535 (30.305)	0.666
Wakefulness after sleep onset	37.382 (36.551)	46.858 (44.678)	27.907 (23.604)	0.102
Total sleep time	375.684 (72.231)	361.617 (78.749)	389.752 (63.964)	0.223
Sleep efficiency	79.558 (12.294)	77.054 (14.572)	82.062 (9.205)	0.202
Hospital Anxiety and Depression Scale				
Anxiety	7.65 (3.711)	7.85 (3.543)	7.45 (3.953)	0.738
Depression	6.85 (3.446)	6.60 (3.299)	7.10 (3.655)	0.652
Epworth Sleepiness Scale	12.33 (5.264)	10.40 (4.784)	14.25 (5.118)	0.019
Satisfaction with Life Scale	20.00 (5.875)	19.55 (6.278)	20.45 (5.568)	0.634
Multidimensional Fatigue Inventory				
MFI-general	13.17 (3.411)	13.65 (3.014)	12.70 (3.785)	0.385
MFI-physical	11.88 (1.362)	12.35 (1.182)	11.40 (1.392)	0.025
MFI-mental	11.70 (3.360)	11.50 (3.253)	11.90 (3.538)	0.712
MFI-activities	12.20 (3.314)	12.20 (3.205)	10.20 (3.503)	1.000
MFI-motivation	11.30 (2.366)	10.70 (2.203)	11.90 (2.426)	0.110
MFI-total score	60.25 (10.744)	60.40 (10.096)	60.10 (11.616)	0.931

Abbreviations. SD: standard deviation; FIR: far infrared; MFI: Multidimensional Fatigue Inventory; BMI: body mass index; SBP: systolic blood pressure; DBP: diastolic blood pressure. ^a^ Data are presented as means (SD) or numbers (%). ^b^ Independent *t*-test or chi-square was used for comparison. As all differences were due to randomization (i.e. chance), *p* values are for reference only.

**Table 2:** Intervention effects on sleepoutcomes across study time-points obtained using the GEE.

Time Points	Time Effect				Time × Group Effect	*p*-Value ^d^
	FIR-Pajamas Group (*n* = 20)		Sham-Pajamas Group (*n* = 20)			
	Mean (SE) ^a^	*Cohen’s d* ^b^	Mean (SE)	*Cohen’s d* ^b^	*d_ppc2_* ^c^	
Pittsburgh Sleep Quality Index						0.439
Baseline	9.50 (0.712)		9.15 (0.634)			
Week 2	8.99 (0.804)	0.15	7.43 (0.536)	0.655	−0.393	0.112
Week 4	8.31 (0.748)	0.364	6.95 (0.628)	0.78	−0.328	0.273
Week 6	8.34 (0.743)	0.356	7.28 (0.461)	0.754	−0.231	0.255
Insomnia Severity Index						0.161
Baseline	14.30 (0.922)		15.10 (1.049)			
Week 2	13.10 (1.054)	0.271	12.29 (1.053)	0.598	−0.357	0.167
Week 4	11.09 (0.993)	0.749	10.30 (0.993)	1.051	−0.353	0.254
Week 6	10.65 (0.930)	0.881	11.30 (1.332)	0.709	−0.033	0.917
Seven-Day Sleep Diary						
Sleep onset latency						0.565
Baseline	36.614 (5.810)		40.535 (6.605)			
Week 6	31.752 (6.173)	0.181	31.973 (4.273)	0.344	−0.13	0.565
Wakefulness after sleep onset						0.176
Baseline	46.858 (9.737)		27.907 (5.144)			
Week 6	24.052 (4.727)	0.666	16.715 (3.103)	0.589	0.327	0.176
Total sleep time						0.852
Baseline	361.617 (17.162)		389.752 (13.941)			
Week 6	393.220 (18.307)	0.398	418.719 (11.630)	0.505	0.037	0.852
Sleep efficiency						0.648
Baseline	77.054 (3.175)		82.062 (2.006)			
Week 6	81.318 (3.004)	0.308	85.219 (1.536)	0.395	0.091	0.648

Abbreviations: FIR, far infrared; SE, standard error; ^a^ estimated mean and standard error (SE) determined with the generalized estimating equation (GEE); ^b^ effect size calculation was based on the difference in the estimated mean and standard deviation when comparing each time point and baseline; ^c^ effect size based on the mean pre–post change in the treatment group minus the mean pre–post change in the control group divided by the pooled pretest standard deviation (Morris, 2008) [36]; ^d^ *p*-value for group × time interaction for mean score using linear mixed-effects models.

**Table 3:** Intervention effects on other outcomes across study time-points obtained using the GEE.

Time Points	Time Effect				Time × Group Effect	*p*-Value ^d^
	FIR-Pajamas Group (*n* = 20)		Sham-Pajamas Group (*n* = 20)			
	Mean (SE) ^a^	*Cohen’s d* ^b^	Mean (SE)	*Cohen’s d* ^b^	*d_ppc2_* ^c^	
HADS Anxiety						0.417
Baseline	7.85 (0.772)		7.45 (0.862)			
Week 2	8.32 (0.762)	−0.137	7.15 (1.095)	0.068	−0.206	0.460
Week 4	6.99 (0.805)	0.244	7.40 (0.878)	0.013	0.217	0.472
Week 6	6.92 (0.805)	0.264	6.66 (0.869)	0.204	0.037	0.891
HADS Depression						0.767
Baseline	6.60 (0.719)		7.10 (0.797)			
Week 2	7.51 (0.970)	−0.238	7.22 (0.833)	−0.033	−0.228	0.435
Week 4	7.42 (1.198)	−0.186	6.66 (0.926)	0.114	−0.364	0.306
Week 6	7.54 (0.958)	−0.57	7.07 (1.050)	0.007	−0.280	0.391
Epworth Sleepiness Scale						0.042
Baseline	10.40 (1.043)		14.25 (1.116)			
Week 2	10.57 (1.135)	−0.035	11.98 (1.224)	0.433	−0.495	0.046
Week 4	9.23 (1.032)	0.252	10.34 (1.267)	0.732	−0.556	0.041
Week 6	7.49 (1.346)	0.54	10.31 (1.139)	0.781	−0.209	0.532
Satisfaction with Life Scale						0.364
Baseline	19.55 (1.368)		20.45 (1.213)			
Week 2	19.26 (1.392)	−0.047	20.60 (1.388)	−0.026	−0.075	0.694
Week 4	20.19 (1.690)	0.093	20.04 (1.404)	0.07	0.178	0.454
Week 6	19.90 (1.549)	0.054	18.94 (1.646)	0.234	0.315	0.179
Multidimensional Fatigue Inventory						
MFI-general						0.328
Baseline	13.65 (0.657)		12.70 (0.825)			
Week 2	14.25 (0.594)	−0.214	12.16 (0.665)	0.161	−0.335	0.264
Week 4	13.20 (0.585)	0.162	12.15 (0.577)	0.173	−0.029	0.908
Week 6	13.31 (0.603)	0.121	12.94 (0.690)	−0.071	0.17	0.635
MFI-physical						0.106
Baseline	12.35 (0.258)		11.40 (0.303)			
Week 2	11.83 (0.361)	0.371	12.11 (0.312)	−0.516	0.958	0.031
Week 4	12.21 (0.398)	0.093	12.34 (0.372)	−0.62	0.841	0.075
Week 6	12.11 (0.504)	0.134	12.31 (0.350)	−0.622	0.896	0.123
MFI-mental						0.906
Baseline	11.50 (0.709)		11.90 (0.771)			
Week 2	12.27 (0.677)	−0.248	12.44 (0.778)	−0.156	−0.068	0.816
Week 4	11.47 (0.661)	0.01	11.35 (0.726)	0.164	−0.154	0.476
Week 6	12.07 (0.786)	−0.17	12.39 (0.805)	−0.139	−0.024	0.947
MFI-activities						0.715
Baseline	12.20 (0.699)		12.20 (0.764)			
Week 2	12.84 (0.716)	−0.202	12.28 (0.591)	−0.026	−0.168	0.408
Week 4	12.31 (0.789)	−0.033	12.38 (0.644)	−0.057	0.021	0.901
Week 6	12.85 (0.695)	−0.209	13.45 (0.695)	−0.383	0.18	0.548
MFI-motivation						0.671
Baseline	10.70 (0.480)		11.90 (0.529)			
Week 2	11.61 (0.586)	−0.38	12.20 (0.546)	−0.125	−0.265	0.447
Week 4	11.38 (0.562)	−0.291	11.85 (0.450)	0.023	−0.317	0.287
Week 6	11.77 (0.711)	−0.394	12.20 (0.511)	−0.129	−0.334	0.372

Abbreviations: FIR, far infrared; SE, standard error; MFI, Multidimensional Fatigue Inventory; ^a^ estimated mean and standard error (SE) determined with the generalized estimating equation (GEE); ^b^ effect size calculation was based on the difference in the estimated mean and standard deviation when comparing each time point and baseline; ^c^ effect size based on the mean pre–post change in the treatment group minus the mean pre–post change in the control group divided by the pooled pretest standard deviation (Morris, 2008) [36]; ^d^ *p*-value for group × time interaction for mean score using linear mixed-effects models.

## Data Availability

The data presented in this study are available on request from the corresponding author. The data are not publicly available due to privacy reasons.

## Supplementary Materials

The following supporting information can be downloaded at: https://www.mdpi.com/article/10.3390/ijerph20053870/s1. Table S1. Intervention effects on PSQI across study time points using GEE (adherent participants).
