# Supplementary material for: Improving Sleep with Far-Infrared-Emitting Pajamas: A Pilot Randomized Controlled Trial
Source: Int J Environ Res Public Health. 2023 Feb 22;20(5):3870. doi: 10.3390/ijerph20053870 (PMC10002002; doi:10.3390/ijerph20053870)
Supplement: Supplementary file 1 [file ijerph-20-03870-s001.zip › ijerph-1995032-supplementary.pdf]

Table S1. Intervention effects on PSQI across study time points using GEE (adherent participants).

Time Points	Time effect				Time × group effect	p-value [e]
	FIR pyjamas group (n = 19)		Sham-pyjamas group (n = 16)			
	**Mean (SE)** [a]	***Cohen's d*** [b]	**Mean (SE)**	***Cohen's d*** [b]	d_{ppc2} [d]	
	Pittsburgh Sleep Quality Index					0.385
Baseline	9.37 (0.738)		9.13 (0.765)			
Week 2	9.11 (0.849)	0.075	7.31 (0.611)	0.657	−0.484	0.089
Week 4	8.28 (0.781)	0.329	6.75 (0.658)	0.834	−0.401	0.197
Week 6	8.17 (0.764)	0.367	7.13 (0.499)	0.774	−0.248	0.243

Abbreviations: FIR, far infrared; SE, standard error; [a] Estimated mean and standard error (SE) from generalized estimating equations (GEE). [b] Effect size calculation was based on the difference of estimated mean and standard deviation comparing each timepoint and baseline. [c] Effect size calculation was based on the between-group difference in total scores divided by pooled standard deviation. [d] Effect size based on the mean pre-post change in the treatment group minus the mean pre-post change in the control group, divided by the pooled pretest standard deviation (Morris, 2008). [e] p-value for group × time interaction of mean score using linear mixed-effects models.
